# Trust in Organ Allocation Systems and Attitudes Toward Organ Donation Among Patients With End-Stage Renal Disease and Their Relatives: A Cross-Sectional Study

**DOI:** 10.7759/cureus.111556

**Published:** 2026-06-26

**Authors:** Giasar Chasan, Evanthia Tsalkidou, Ilker Memet, Sempaetin Gkasil, Lana Kalaitsidou, Gregory Trypsianis, Christos Argyriou, Christos Tsalikidis

**Affiliations:** 1 Surgery, General Hospital of Komotini, Komotini, GRC; 2 Emergency, General Hospital of Komotini, Komotini, GRC; 3 Medical Statistics, Democritus University of Thrace, Alexandroupolis, GRC; 4 Vascular Surgery, Democritus University of Thrace, Alexandroupolis, GRC; 5 General Surgery, University General Hospital of Alexandroupolis, Alexandroupolis, GRC

**Keywords:** dialysis, end-stage renal disease, healthcare trust, health literacy, health policy, organ allocation, organ donation, transplantation

## Abstract

Background: Trust in healthcare systems is an important factor in patient decision-making and attitudes toward organ donation. Objective: This study aims to examine the association between trust in organ allocation systems and attitudes toward organ donation, willingness to donate, knowledge, and concerns regarding fairness and transparency among patients with end-stage renal disease (ESRD) and their relatives. Materials and methods: A cross-sectional questionnaire-based study was conducted between December 2020 and September 2021 at two tertiary healthcare centers in Greece. Participants were recruited from dialysis units and outpatient nephrology clinics. A total of 178 individuals participated, including 104 patients with ESRD and 74 first-degree relatives. Patients and relatives were analyzed as independent groups. The questionnaire was adapted from previously published studies assessing attitudes toward organ donation, trust in healthcare systems, and transplantation-related decision-making. The instrument was reviewed for content relevance, pilot-tested before administration, and revised for clarity, although it was not formally validated. Data were collected through researcher-administered structured interviews. The questionnaire assessed trust in organ allocation systems, attitudes toward organ donation, willingness to donate, knowledge, and demographic characteristics. Higher education was defined as completion of post-secondary, university, or postgraduate education. Participants responding “no” to the question “Do you trust the organ allocation system?” were classified as having limited trust.

Continuous variables are presented as mean ± standard deviation, and categorical variables as frequencies and percentages. Associations between categorical variables were assessed using chi-square tests. No substantial missing data were identified, and all completed questionnaires were included in the analysis. No adjustment for multiple comparisons was performed because of the exploratory nature of the study. Data were analyzed using SPSS Statistics version 26 (IBM Corp. Released 2019. IBM SPSS Statistics for Windows, Version 26.0. Armonk, NY: IBM Corp.), and statistical significance was set at p < 0.05.

Results: A total of 46% of participants reported limited trust in organ allocation systems. Lower trust was significantly associated with reduced willingness to donate organs (p = 0.02) and increased concerns regarding fairness and transparency (p = 0.01). Relatives reported lower levels of trust than patients (52% vs. 41%, p = 0.04).

Conclusions: Trust in organ allocation systems was closely associated with attitudes toward organ donation and willingness among patients with ESRD and their relatives. Relatives reported lower levels of trust in organ allocation systems than patients did. Strengthening transparency, communication, and institutional credibility may be associated with greater willingness to donate and improved engagement with transplantation systems. These findings may help inform trust-based strategies and policy interventions aimed at improving organ donation systems.

## Introduction

For many patients with end-stage organ failure, organ transplantation represents the preferred treatment option. Despite advances in transplantation medicine, the persistent shortage of donor organs remains a major global health challenge. Worldwide, only a fraction of the demand for transplantable organs is met each year, leaving many patients on waiting lists and contributing to preventable morbidity and mortality [1,2].

Beyond clinical and logistical barriers, social and psychological factors, including religious beliefs, cultural norms, altruism, and trust in healthcare institutions, play an important role in determining organ donation behavior. Among these, trust in healthcare systems, particularly in organ allocation systems, has emerged as a central factor in attitudes toward organ donation and decision-making processes [3-6].

In the context of organ donation, trust may be defined as the expectation that healthcare institutions and organ allocation systems will act competently, fairly, transparently, and in the best interests of patients. Although related, trust in healthcare systems and trust in organ allocation systems represent distinct constructs. Trust in healthcare systems reflects confidence in healthcare institutions and professionals more broadly. In contrast, trust in organ allocation systems specifically concerns perceptions of fairness, transparency, and legitimacy in the allocation of donor organs. Distinguishing among these forms of trust is important because concerns about allocation procedures may be associated with attitudes toward organ donation, even among individuals who generally trust healthcare services.

Trust is closely associated with perceptions of fairness, transparency, and legitimacy in organ allocation systems. Individuals who perceive allocation processes as opaque or inequitable may report lower willingness to participate in organ donation programs [6-8]. Conversely, higher levels of trust have been associated with greater willingness to donate and higher engagement with healthcare systems [7-9].

Previous studies have demonstrated that distrust in healthcare institutions is associated with lower donation rates and greater reluctance to consent [8-10]. However, much of the existing literature has been conducted in general populations, primarily in Western countries, and relatively few studies have focused specifically on patients with end-stage renal disease (ESRD) and their relatives. Furthermore, most available studies employ cross-sectional designs, limiting the ability to assess changes in trust and donation attitudes over time. Additionally, disparities in access to information and health literacy may be associated with differences in trust and decision-making. These gaps highlight the need for further investigation in populations directly affected by organ shortage and transplantation-related decisions.

Patients with ESRD and their relatives represent a critical population, as they directly experience the consequences of organ shortage. Their perceptions may be associated with individual decisions and broader system outcomes. In numerous organ donation contexts, family members are often involved in discussions about the donation process. They may be asked to provide consent for organ donation, making their attitudes and level of trust particularly important.

Although previous studies have examined attitudes toward organ donation in the general population, limited evidence is available regarding trust in organ allocation systems among patients with ESRD and their relatives. This population is uniquely positioned to provide insight into trust-related factors due to its direct experience with transplantation decisions and organ shortages. By focusing on both patients and their relatives, the present study addresses an important gap in the literature. It contributes evidence that may help inform trust-based interventions and organ donation policies.

The present study examines the association between trust in organ allocation systems and attitudes toward organ donation, willingness to donate, and concerns regarding fairness and transparency among patients with ESRD and their relatives.

Research questions

This study examined whether trust in organ allocation systems is associated with attitudes toward organ donation, willingness to donate, and concerns about fairness and transparency among patients with ESRD and their relatives. We hypothesized that (1) lower trust in organ allocation systems would be associated with reduced willingness to donate organs and (2) relatives would report lower trust levels than patients with ESRD. These findings have significant implications for health policy and public trust in transplantation systems.

## Materials and methods

Study design, setting, and participants

A cross-sectional questionnaire-based study was conducted at a tertiary healthcare center. A total of 178 individuals participated, including 104 patients with ESRD undergoing dialysis and 74 relatives. Participants were recruited using a convenience sampling method.

Eligibility criteria

Participants were eligible for inclusion if they were aged ≥18 years, had a confirmed diagnosis of ESRD (for patients), and provided informed consent. Individuals were excluded if they had documented cognitive impairment based on available medical records or clinical judgment at the time of recruitment or if they declined to participate in the study.

Data collection

No additional exclusion criteria were applied to family members. Data were collected using a structured questionnaire designed to assess participants’ trust in healthcare systems and organ allocation processes, knowledge of organ donation, and attitudes toward organ donation.

Statistical analysis

Descriptive statistics were used. Associations between variables were analyzed using chi-square (χ²) tests. Data were analyzed using SPSS Statistics version 26 (IBM Corp. Released 2019. IBM SPSS Statistics for Windows, Version 26.0. Armonk, NY: IBM Corp.). Statistical significance was set at p=0.05. No adjustment for multiple comparisons was performed because of the exploratory nature of the study.

Ethical considerations

Ethical approval was obtained from the Scientific Council of the University General Hospital of Alexandroupolis (approval no. 15/20-01-2020) and the Board of Directors of the General Hospital of Komotini (Sismanoglio) (decision no. 120/13-02-2020). Participation was voluntary, and written informed consent was obtained from all participants by a member of the research team prior to questionnaire administration at the participating dialysis units and outpatient nephrology clinics. Participants were informed that they could withdraw from the study at any time without any consequences. No financial compensation, incentives, or other benefits were provided to participants for their participation in the study. Participant confidentiality was maintained throughout the study. Questionnaires were anonymized prior to analysis, and all collected data were stored securely with access restricted to the research team. The study was conducted in accordance with the principles of the Declaration of Helsinki.

## Results

The demographic characteristics of the study participants are presented in Table 1.

**Table 1:** Demographic characteristics of participants Values are presented as mean ± standard deviation or n (%), as appropriate. ESRD: end-stage renal disease

Characteristic	ESRD patients (n = 104)	Relatives (n = 74)
Mean age, years	58 ± 13	46 ± 15
Male sex	61 (59%)	33 (45%)
Higher education	29 (28%)	31 (42%)
Married	73 (70%)	48 (65%)
Employed	36 (35%)	44 (59%)
Dialysis duration >3 years	57 (55%)	Not applicable

A substantial proportion of participants (46%) reported limited trust in organ allocation systems, defined as responding “no” to the question “Do you trust the organ allocation system?”. Lower levels of trust were significantly associated with reduced willingness to donate organs (p = 0.02) and increased concerns regarding fairness and transparency (p = 0.01). Relatives demonstrated significantly lower levels of trust compared to patients (38/74 (52%) vs. 43/104 (41%), p = 0.04). Trust-related attitudes and willingness to donate organs are summarized in Table 2.

**Table 2:** Trust-related attitudes and willingness toward organ donation Values are presented as n (%). ESRD: end-stage renal disease

Variable	ESRD patients (%) (n = 104)	Relatives (%) (n = 74)	p-value
Limited trust in allocation systems, n (%)	43 (41%)	38 (52%)	0.04
Willingness to donate organs, n (%)	66 (63%)	33 (45%)	0.02
Concerns regarding fairness and transparency, n (%)	40 (38%)	42 (57%)	0.01

## Discussion

The findings highlight trust as a key structural factor associated with organ donation behavior. Trust is not merely an individual attitude but reflects broader perceptions of institutional legitimacy and fairness.

Policy implications

These findings highlight the importance of integrating trust-building strategies into organ donation policies. Public health interventions may include improving public access to information regarding organ allocation procedures and allocation criteria, strengthening communication between healthcare professionals and patients, and implementing targeted educational initiatives for patients with ESRD and their relatives. Policymakers may also consider monitoring trust-related indicators using regular surveys, such as annual assessments of patient and family perceptions regarding fairness, transparency, and confidence in organ allocation procedures, and incorporating these measures into national transplantation strategies. The association between low trust and concerns about fairness underscores the importance of procedural justice. Transparent allocation systems are essential for maintaining public confidence [11].

Knowledge and trust

The observed association between knowledge and trust suggests that informational factors may be related to confidence in healthcare systems. However, because of the cross-sectional design, the direction of this association cannot be determined. It cannot be established whether greater knowledge is associated with higher trust or whether individuals with higher trust are more likely to seek information and report greater knowledge. Further research is needed to clarify the relationship between knowledge, trust, and attitudes toward organ donation [12,13].

Differences between patients and relatives

The observed differences between patients and relatives may reflect variations in exposure to the healthcare system [14,15]. Patients, through direct interaction, may report greater trust, whereas relatives may rely more on external perceptions and uncertainties. However, demographic differences between the groups, including age, educational level, and employment status, may also have contributed to the observed differences in trust and should be considered as potential confounding factors. Previous studies have emphasized the importance of public trust, transparency, and institutional legitimacy in maintaining effective organ donation systems and public engagement [16-19].

Limitations

Because of the cross-sectional design, the direction of the observed associations cannot be determined. Although lower trust was associated with reduced willingness to donate organs, it cannot be established whether limited trust is related to donation attitudes or whether pre-existing attitudes toward organ donation are related to trust in healthcare systems. Longitudinal studies are required to clarify the temporal relationship between these factors.

This study has several limitations, including its cross-sectional design, use of convenience sampling, and reliance on self-reported data. The single-center setting may limit the generalizability of the findings. Additionally, the absence of multivariable regression analysis limited the ability to account for potential confounding variables. Selection bias may also have affected the composition of the study sample and the reported responses.

Furthermore, the relatively small sample size may have limited the statistical power to detect additional associations. The exact response rate could not be calculated because the number of individuals approached for participation was not systematically recorded. Additionally, no a priori sample size calculation or power analysis was performed because the study used a convenience sampling approach. Effect size measures, such as Cramer's V or odds ratios, were not calculated, limiting the interpretation of the magnitude of the observed associations. Moreover, no adjustment for multiple comparisons was performed; therefore, the possibility of Type I error resulting from multiple statistical tests cannot be excluded. Although patients and relatives were analyzed as independent groups, some family members may have been related to participating patients, and the potential effect of intra-family clustering could not be assessed.

Additionally, the questionnaire was adapted from previously published studies but was not formally validated. Social desirability bias may have influenced responses because attitudes toward organ donation were self-reported during researcher-administered interviews. Furthermore, potentially important factors such as religious beliefs, cultural influences, and prior exposure to organ donation or transplantation were not assessed. They may be associated with participants’ attitudes and levels of trust.

## Conclusions

Trust in organ allocation systems was closely associated with attitudes toward organ donation and willingness among patients with ESRD and their relatives. Relatives reported lower levels of trust in organ allocation systems than patients did. Transparency, communication, and institutional credibility were associated with attitudes toward organ donation and willingness to donate within the study population. These findings highlight the importance of trust-related factors in organ donation systems and may help inform future research, policy discussions, and transplantation strategies.
